# Case Report: Whole genome sequencing identifies *CCDC88C* as a novel *JAK2* fusion partner in pediatric T-cell acute lymphoblastic leukemia

**DOI:** 10.3389/fped.2022.1082986

**Published:** 2023-01-10

**Authors:** Aleksandra Krstic, Fatemah Rezayee, Leonie Saft, Anna Hammarsjö, Petter Svenberg, Gisela Barbany

**Affiliations:** ^1^ Clinical Genetics, Karolinska University Hospital, Stockholm, Sweden; ^2^ Department of Molecular Medicine and Surgery, Karolinska Institutet, Stockholm, Sweden; ^3^ Clinical Pathology and Cancer Diagnostics, Karolinska University Hospital, Stockholm, Sweden; ^4^ Department of Clinical Pathology and Oncology, Karolinska Institute, Stockholm, Sweden; ^5^ Pediatric Oncology, Karolinska University Hospital, Stockholm, Sweden; ^6^ Department of Women’s and Children’s Health, Karolinska Institutet, Stockholm, Sweden

**Keywords:** pediatric T-ALL, whole genome sequencing, JAK2 fusions, targeted therapy, precision medicine

## Abstract

In the present report, we applied whole genome sequencing (WGS) to genetically characterize a case of pediatric T-cell acute lymphoblastic leukemia (ALL) refractory to standard therapy. WGS identified a novel *JAK2* fusion, with *CCDC88C* as a partner. *CCDC88C* encodes a protein part of the Wnt signaling pathway and has previously been described in hematological malignancies as fusion partner to *FLT3* and *PDGFRB*. The novel *CCDC88C*::*JAK2* fusion gene results in a fusion transcript, predicted to produce a hybrid protein, which retains the kinase domain of JAK2 and is expected to respond to JAK2 inhibitors. This report illustrates the potential of WGS in the diagnostic setting of ALL.

## Introduction

Current treatment protocols for pediatric acute lymphoblastic leukemia (ALL) use information regarding genomic aberrations in the leukemic blasts together with clinical features at presentation, and initial therapy response is used to assign patients to different risk categories and treatment intensities (1). Survival of pediatric acute ALL in risk-adapted chemotherapy trials exceeds 90% (2); however, further improvements in outcome are not likely to be achieved with conventional chemotherapy but will require alternative treatment approaches such as immunotherapy or targeted therapy.

High throughput sequencing technologies have shown enormous potential in the genetic characterization of hematological malignancies, particularly in pediatric ALL (3), and are paving the way to personalized treatment strategies, although implementation in clinical practice is still meager (4). Technologies that interrogate the whole genome with high resolution are increasingly being adopted in the diagnostic workout of pediatric ALL (5). Here, we report a novel *JAK2* fusion in a pediatric case of therapy-refractory T-ALL that was detected using paired-end whole genome sequencing (WGS) in the diagnostic setting.

## Case presentation

A 7-year-old boy presenting with a leukocyte count 800 × 10^9^/L and peripheral lymphadenopathy was diagnosed with precursor T-ALL with extensive involvement of the bone marrow (BM) and peripheral blood. Multiparameter flow cytometry showed an immature T-lymphoblast population (95%) of cortical phenotype (EGIL III; CD45dim/CD7+/cytCD3+/Tdt+//CD4/CD8dim+/CD10−/CD1ahetero/CD56/16−/CD99++/CD5dim+/ CD2+/CD48+/CD38+//TCRa/b−/TCRg/d−/HLA−DR− {Bene, 1995 #41}). The blasts were negative for myeloid markers (cytMPO and CD33) and for B-cell markers (CD19 and cytCD79a). According to the standard of care (SoC) genomic characterization, the patient had a normal karyotype and none of the targeted fluorescence *in situ* hybridization (FISH) analysis was positive, i.e., no *KMT2A*- or *BCR::ABL* rearrangements and no *ABL*-class rearrangement were detected. A biallelic *CDKN2A/B* deletion was the only clonal aberration detected. The patient responded poorly to standard four-drug induction (dexamethasone, pegylated-asparaginase, daunorubicin, and vincristine) and was switched to consolidation at day 19 (6-mercaptopurine, cytarabine, and cyclophosphamide). Two weeks later, he still had 80% blasts in the bone marrow and was considered refractory and treatment with nelarabine initiated. Following two rounds of nelarabine and one additional high-risk chemotherapy block, remission was achieved, and he subsequently underwent hematopoietic stem cell transplantation (HSCT) from his haploidentical mother. The conditioning regimen consisted of ATG (grafalon), fludarabine, and thiotepa together with total body irradiation (TBI) (12 Gy in four fractions) and rituximab after which he received an alpha/beta T-cell-depleted peripheral stem cell graft. Initially, mycophenolate mofetil was switched to cyclosporin together with prednisolone after 45 days due to acute gut and skin graft versus host disease (GVHD). He is currently 8 months post HSCT, with no sign of disease but still on a low dose prednisolone.

At the time of initial failure, the genetic laboratory was engaged to screen for potential targets for experimental therapy. DNA isolated from the bone marrow sample taken at diagnosis was sequenced using a PCR-free, paired-end WGS protocol with a 30× coverage on an Illumina HiSeq X platform at Clinical Genomics (SciLifeLab, Stockholm) and annotated to the Human GRCh37 (hg19) build. Variants were identified using the MIP (6) validated for routine diagnostics and visualized in the SCOUT interface (7). MIP performs structural variant (SV) detection using Delly (8), TIDDIT V2.0 (9), as well as Manta (10) and single nucleotide variant (SNV) detection using GATK HaplotypeCaller (11). Subsequently, filtering was performed using the list of targetable genes described in the INFORM study (12). This analysis resulted in 260 SNVs and 18 SVs. Further filtering of the SNVs based on gnomAD or local observations as well as annotations in ClinVar narrowed the list to 11 SNVs that were manually inspected and dismissed, as no targetable SNV was identified. Similarly, the 11 SVs were manually curated based on recurrency in the local database and inspection in Integrated Genomic Viewer (IGV) (13) leaving only three SVs that interestingly affected regions recurrently involved in ALL.

**Figure 1:**
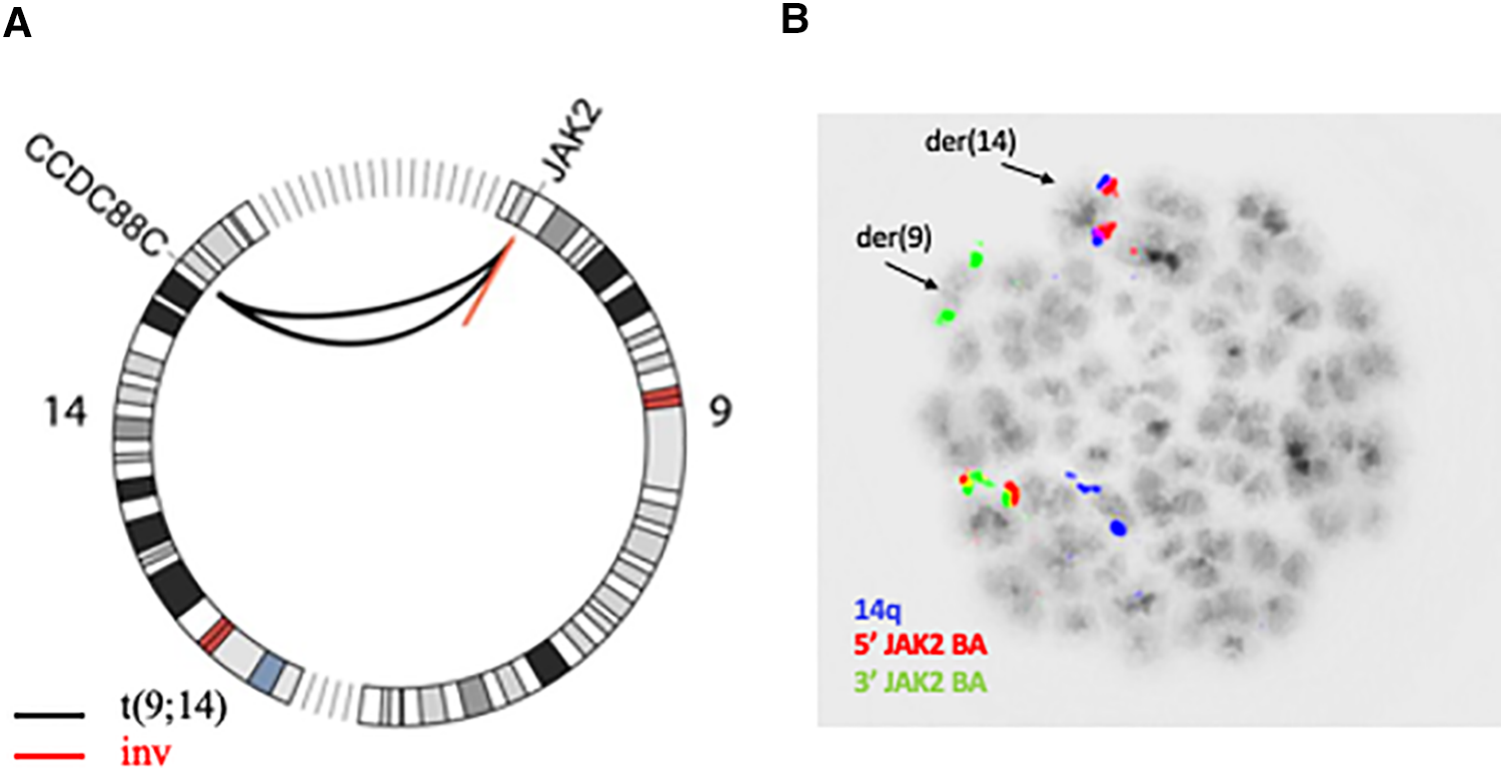
(**A**) Circos plot showing chromosomes 9 and 14; the black lines represent the reciprocal translocation together with the concomitant inversion of the small fragment from 9p23.1 (red line). (**B**) Aberrant metaphase hybridized with *JAK2* break apart probe (red/green signal, CytoTest) and 14 centromere probe (blue signal) showing the distal red signal (5′ end) of *JAK2* translocated to 14q.

Two of these SVs affected the *JAK2* locus on 9p24.1 and inspection in IGV revealed a shared breakpoint suggesting that both SVs had occurred in the same genomic rearrangement (Supplementary Figure 1). The first SV consisted in a 122 kb-long intrachromosomal inversion with breakpoints that mapped to intron 15 (NM_001322194.2) and telomeric to the *JAK2* locus. The second SV consisted in an interchromosomal event and shared the breakpoint in intron 15 of *JAK2*, while the second breakpoint was located at 14q32.11 and mapped to intron 25 in the *CCDC88C* locus (NM_001080414). Both events are visualized in the Circos plot in Figure 1A. The rearrangement of *JAK2* could be verified with metaphase FISH that confirmed that the 5′ signal at the JAK2 locus was translocated to chromosome 14 while the 3′ signal was retained on derivative chromosome 9 (Figure 1B). As the rearrangement involves the most distal regions of chromosomal arms 9p and 14q, it is beyond the resolution of chromosome banding analysis and cannot be detected by karyotype analysis. In addition, SV analysis also detected a deletion on 9p21.3 encompassing 39 kb and supported by 95% of reads that resulted in a biallelic loss of *CDKN2A/B* that had been previously found in SoC testing.

**Figure 2:**
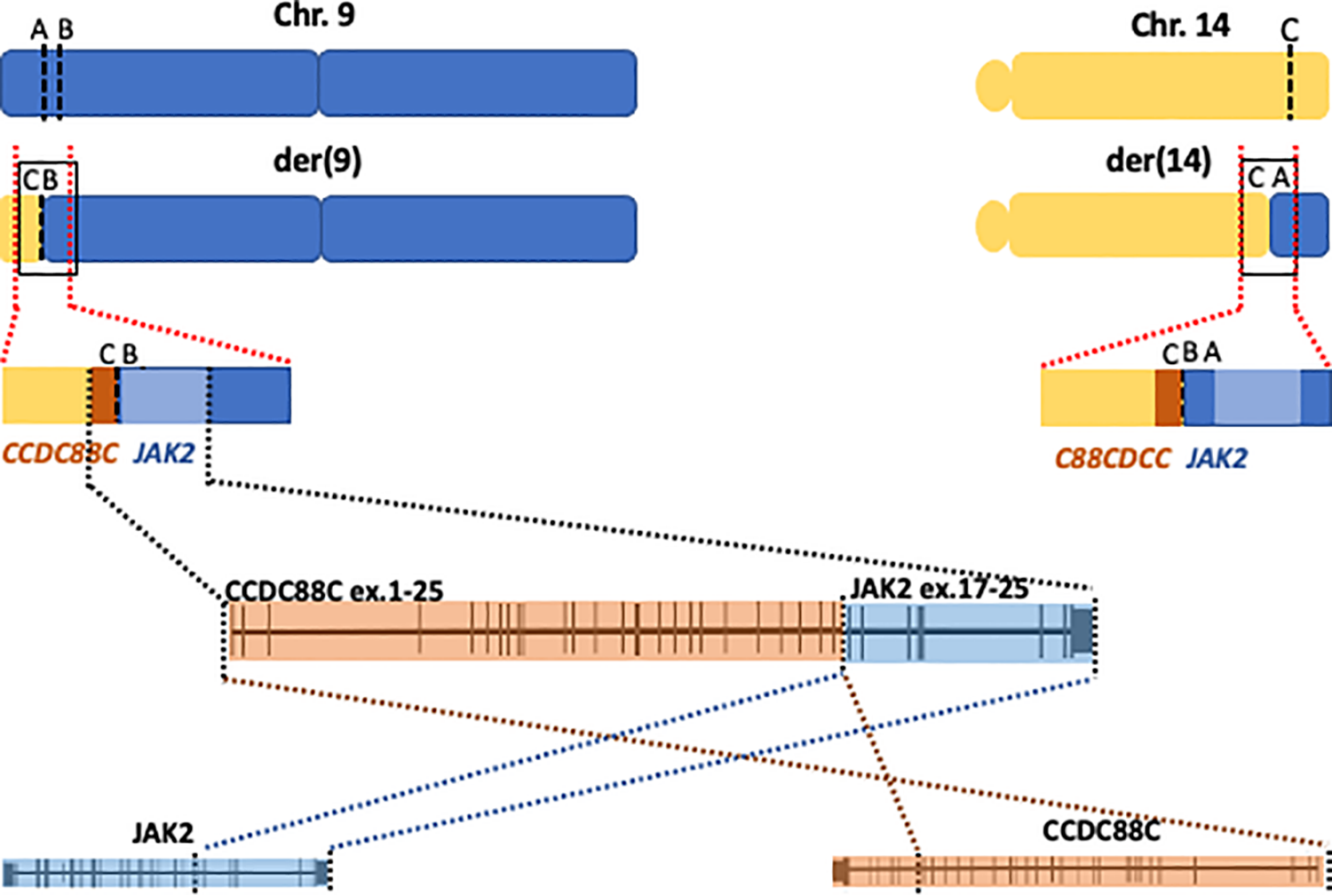
Cartoon showing a schematic representation of chromosomes 9 (blue) and 14 (yellow) at the derivative chromosomes 9 and 14 (upper panel), the black squares mark the junctions. Middle cartoon represents a blow up of the junctions illustrating the creation of the fusion gene on derivative chromosome 9 (der9). The lower panel represents a magnification of the fusion gene showing exons 1–25 in *CCDC88C* joined to exons 16–25 in *JAK2*.

Taken together the data indicated a reciprocal translocation t(9;14)(p24.1;q32.11) with a concomitant inversion of a 122-kb-long fragment from 9p that resulted in the creation of a fusion gene joining intron 25 of *CCDC88C* to intron 15 of *JAK2* on derivative chromosome 9. As a result of the inversion from the 9p fragment, the genes are arranged in opposite orientations and thus no reciprocal fusion is created at derivative 14 (Figure 2).

According to the WGS findings, the rearrangement juxtaposes exons 1 to 25 of *CCDC88C* to exons 16 through 25 of *JAK2*, creating a novel fusion gene (Figure 2). The expression of the fusion transcript joining exon 25 of *CCDC88C* (3′ partner) to exon 16 in *JAK2* gene (5′ partner) was confirmed with RT-PCR followed by Sanger sequencing. The fusion gene will thus result in an in-frame hybrid protein with the kinase domain of JAK2 at the carboxy-terminal.

## Discussion

*JAK2* encodes a nonreceptor tyrosine kinase involved in cell proliferation and differentiation through the JAK/STAT signaling pathway. Aberrant *JAK2* is found in several cancers including both myeloid and lymphoid malignancies (14). Fusions involving *JAK2* were found in Philadelphia-like ALL (15), a subset of high-risk B-ALL characterized by a distinctive gene expression profile resembling Philadelphia-positive ALL, but lacking the canonical *BCR::ABL* fusion (16, 17). A fraction of Philadelphia-like ALL harbors *JAK2* fusions where *JAK2* is the 5′ partner and all the fusions described so far include exons 19–25 and retain the kinase domain that is constitutively activated in the fusions (18). To this date, over 30 genes have been found as potential 3′ partners to *JAK2*, but this is the first report of a *CCDC88C*::*JAK2* fusion. JAK2 inhibitors have shown antileukemic effect in *ex vivo* experiments in cells carrying *JAK2* fusions (19, 20), that include the kinase domain of JAK2; however, the experience *in vivo* is limited (21). *JAK2* fusions have only been described in isolated cases of T-ALL (18), although activating point mutations in members of the JAK/STAT pathway has been reported in up to 25% of patients with T-ALL (22). Patients carrying genomic alterations that activate the JAK/STAT pathway either through gain of function mutations or gene fusion that include the JAK2 kinase domain are potential candidates for targeted therapy (23) (24). Pediatric patients with ALL and genomic features consistent with JAK/STAT pathway activation are currently recruited in ongoing trials (clinicaltrials.gov NCT03117751 and NCT02723994) for targeted treatment with ruxolitinib (selective JAK1/2 inhibitor) in addition to chemotherapy. Since our patient responded to nelarabine, he was never considered for therapy with JAK inhibitors nor were cells available to perform *in vitro* sensitivity tests.

*CCDC88C* is a ubiquitous protein, involved in the Wnt signaling pathway, with multiple functions that include determination of cell polarity, development of the nervous system, and tumor suppressor activity (25). CCDC88C protein is highly expressed in B- and T-cell pediatric leukemias (https://pecan.stjude.cloud/proteinpaint/CCDC88C). *CCDC88C* has been reported a handful occasions in hematological malignancies as fusion partner with *FLT3* (26) as well as *PDGFRB* (27) including a case report of pediatric patient with a myeloproliferative disorder (28). It has also been reported as the 5′ fusion partner to PDGFRB in a young adult with Philadelphia-like ALL that responded to imatinib (29).

The genetic landscape of T-ALL is widely heterogeneous, and despite several recurrent aberrations, to date no single lesion is used to risk-stratify T-ALL patients. However, certain aberrations such as BCR-ABL and other ABL-class fusions or KMT2A rearrangements, although rare, contribute important information to individualized patient management.

Since our patient responded to nelarabine, he could be bridged to transplantation and experimental therapy with JAK2 inhibitors was not considered. However, this report illustrates the power of WGS in the diagnostic setting of ALL and describes a novel *CCDC88C*::*JAK2* fusion in T-ALL, adding *CCDC88C* to the list of potential *JAK2* partners. While the validation of WGS in the diagnostic setting of acute leukemias is still ongoing in our laboratory, the present report serves as a real-life example of the power of WGS in the diagnostic setting of acute leukemia, as it enables timely recognition of potential therapeutic targets in high-risk pediatric ALL.

## Data Availability

The raw data supporting the conclusions of this article will be made available by the authors, without undue reservation.
