# Supplementary figures and images for: Case Report: Whole genome sequencing identifies CCDC88C as a novel JAK2 fusion partner in pediatric T-cell acute lymphoblastic leukemia
Source: Front Pediatr. 2023 Jan 10;10:1082986. doi: 10.3389/fped.2022.1082986 (PMC9871838; doi:10.3389/fped.2022.1082986)

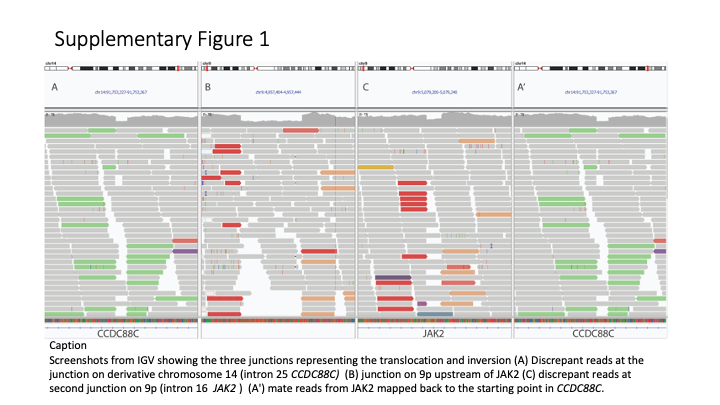

Supplement: Supplementary file 1 [file Image1.tiff]
